# A red-eye presentation concerning for vernal keratoconjunctivitis: a case report

**DOI:** 10.1186/s13223-026-01029-x

**Published:** 2026-04-06

**Authors:** Lauren Wong, Samira Jeimy

**Affiliations:** 1 Department of Medicine, Schulich School of Medicine and Dentistry, Western University, London, ON Canada; 2 Division of Clinical Immunology and Allergy, Western University, London, ON Canada

**Keywords:** Conjunctivitis, Vernal keratoconjunctivitis, Red eye

## Abstract

**Background:**

Vernal keratoconjunctivitis (VKC) is a chronic, recurrent allergic eye disease that can significantly impair quality of life in children. Although rare in Canada and more common in hot, dry climates, VKC should be considered in persistent conjunctivitis. Diagnosis may be challenging when symptoms occur outside typical seasonal patterns or when allergy testing is inconclusive.

**Case presentation:**

An 11-year-old male presented with a six-month history of bilateral conjunctival redness and swelling with seasonal nasal and palatal pruritus and sneezing. Symptoms began atypically in February and progressed to photophobia and early vision changes. Clinical images demonstrated perilimbal Horner–Trantas dots with marked conjunctival inflammation, ecchymosis, and chemosis. Epicutaneous testing and serum IgE showed only mild sensitization to cat dander. Symptoms partially improved with olopatadine and resolved with topical loteprednol, though attempts to taper resulted in recurrence. VKC was diagnosed based on clinical features and photographic findings. The patient was transitioned to olopatadine and treated with oral rupatadine. After ophthalmology assessment and steroid taper, he remained stable on daily antihistamine therapy and later discontinued medications with only mild intermittent ocular symptoms. Repeat skin testing was negative.

**Conclusions:**

VKC is uncommon in Canada, but may lead to vision-threatening complications if a patient is not appropriately seen by an ophthalmologist and it goes unrecognized. It should be considered in cases of conjunctivitis that are unresponsive to antihistamines, even when seasonal patterns or allergen sensitization are unclear. Topical and oral antihistamine combination is effective in stabilizing VKC symptoms, with the addition of topical steroids if needed for flares. VKC treatment can require a prolonged course.

## Background

VKC is a rare allergic inflammatory disease that affects the ocular conjunctiva and cornea. It is thought to be caused by allergen exposure that triggers mast cell activation in conjunctival tissue [1]. Eosinophils have also been implicated, making up the majority of conjunctival secretions in these patients [1]. It most commonly affects males in early to mid-childhood living in hot and dry climates such as the Mediterranean, Middle East, and Central and South America where there is high dust and sunlight exposure [1, 2]. A positive family history of allergic disease is present in 40–50% of cases, and personal history of atopy including asthma, eczema, or rhinitis is present in about 46% of cases [3]. 

VKC is classically characterized by bilateral itchy eyes, tearing, discharge, irritation, redness, and photophobia [4]. Episodes are often recurrent with peak incidence in the spring or summer seasons, and the potential to become perennial [4]. 

We present a case of VKC in Canada with chronic symptoms that developed outside of typical seasonal patterns with no clear provoking allergen. This case highlights clinical features that may help clinicians, particularly allergists and primary care physicians, recognize VKC and consider timely ophthalmology referral when appropriate.

## Case presentation

An 11-year-old male presented to the Allergy and Immunology clinic with a six-month history of bilateral redness and swelling of the conjunctiva. He had experienced seasonal nasal and palatal itch and sneezing from May to October for the last 3–4 years but had no known atopic history other than resolved preschool asthma.

The initial onset of symptoms was in February, however in May of the presenting year he began noticing vision changes and perilimbal white dots were identified by the clinician on photographs shared in the clinic. The findings were consistent with Horner–Trantas dots, a characteristic feature of vernal keratoconjunctivitis. Review of systems was otherwise unremarkable. He tried multiple eyedrops, only experiencing relief with olopatadine eyedrops. Following optometry assessment, he switched to loteprednol steroid eyedrops. He had full resolution of symptoms on loteprednol but flared when attempting to taper off of it. He was still using loteprednol, in addition to ciclesonide and desloratadine nasal sprays as needed at the time of allergy consultation.

**Fig. 1:**
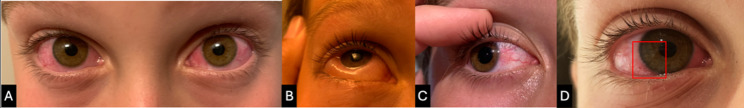
An 11-year-old male with vernal keratoconjunctivitis (VKC), showing conjunctival erythema (**A**), ecchymosis (**B**), chemosis (**C**), and possible Horner-Trantas dots (**D**)

On physical exam, there were no significant findings other than internal nasal changes characteristic of allergic rhinitis. However, photos from one month prior demonstrated significant conjunctival erythema, ecchymosis, and possible Horner-Trantas dots **(**Fig. 1**)**. Epicutaneous testing was positive to cat dander, but no other allergens on the broad aeroallergen panel including dust mite, pet dander, pollens, and molds. Serum IgE was ordered for the same panel to assess for allergens that may not have appeared positive on epicutaneous testing due to young age, with only cat returning mildly elevated.

The patient initially flared without any clear cat or other significant environmental allergen exposures in February, although based on clinical history and photos during flares, the diagnosis of vernal keratoconjunctivitis (VKC) was made. Atopic keratoconjunctivitis and giant papillary conjunctivitis were also considered. He was advised to restart olopatadine eyedrops to bridge towards tapering off the loteprednol, and to use rupatadine orally as needed for both ocular and nasal allergic symptoms.

He was referred to ophthalmology for consideration of alternate management options, given the impacts of his keratoconjunctivitis on his quality of life. When he was seen, he had been off loteprednol eyedrops for one month, with improvement in ocular symptoms. No changes were made to his treatment plan as he was in low disease activity.

He remained stable off loteprednol with no major flares, though continued to have minor conjunctival erythema while on olopatadine one drop daily and rupatadine 5 mL liquid daily. His rupatadine was adjusted from liquid to tablet form and increased to 10 mg oral daily with flexibility to titrate to 40 mg daily. He also continued to use olopatadine once daily. The patient remained on this regimen for one year, with no new ocular symptoms. Repeat epicutaneous testing 20 months after initial diagnosis did not reveal additional environmental allergens. Within seven months he had completely discontinued antihistamine use, with only intermittent pruritus of the eyes and mild daily conjunctivitis that was not bothersome to him. Prescriptions for oral and topical antihistamines were renewed should his vernal keratoconjunctivitis flare again, and follow-up was left open.

### Discussion and conclusions

Although the diagnosis of VKC should consider the history of presenting illness, ocular examination, and investigations, it is largely clinical. Two forms of VKC exist: tarsal which involves the upper tarsal conjunctiva, and limbal which involves the bulbar conjunctiva [2, 3]. In the tarsal form, there may be erythematous and thickened eyelids and papillae on the upper tarsal conjunctiva with a cobblestone appearance [3, 4]. Main findings in the limbal form are gelatinous infiltrations around the limbus surrounding the cornea (Horner-Trantas dots), which are focal collections of degenerated eosinophils and epithelial cells [4]. The mixed form has features of both. In this case, the photographic identification of Horner-Trantas dots strongly aided in the clinical diagnosis of VKC. Corneal involvement can also occur in cases where VKC is not adequately or promptly treated, which is why ophthalmology involvement can be important [5]. It will initially present as punctate epithelial erosions with progression to macro erosions and ulcers, causing gradual vision loss [5]. 

Differential diagnoses include other subtypes of allergic conjunctivitis such as seasonal allergic conjunctivitis, perennial allergic conjunctivitis, atopic keratoconjunctivitis, and giant papillary conjunctivitis [5]. VKC should be suspected when there is failure of treatment with common topical or systemic antihistamines traditionally used in allergic conjunctivitis [5]. Symptoms such as photophobia and ocular pain are more specific for VKC [5]. Not all VKC patients will have positive epicutaneous tests, and the presentation or flares of the disease are not always associated with large environmental allergen exposure [4, 6]. This case presented an example of VKC manifesting without positive initial or repeat epicutaneous testing, and no clear allergen trigger. Approximately 50% of patients with VKC have a negative allergy test, as other non-IgE mechanisms are implicated in the inflammatory reaction of VKC [6]. 

Treatment is a stepwise approach, with the most important step being the removal of any possible allergens in the patient’s environment [4–6]. Conservative management for ocular itching, burning, and irritation includes cold compresses and saline rinses [5]. Antihistamine or mast cell stabilizer eyedrops, or dual acting agents with both can be added, though complete resolution of disease may not be attained due to the abovementioned mixed IgE and non-IgE pathogenesis [4]. Olopatadine topical antihistamine eyedrops are commonly used to target the H1 receptors [4]. Corticosteroid eyedrops such as loteprednol and fluorometholone are effective in disease flares, but are not to be used for long-term management [5]. Ideally steroids are used as pulse therapy with a taper in addition to antihistamine or mast cell stabilizers as maintenance therapy, as was done in this case. In more severe disease with persistent inflammation, steroid dependence, macropapillae, and ulcers or macro erosions, steroid-sparing topical immunomodulators like cyclosporine and tacrolimus are routinely used [4]. Generally, VKC is a self-limiting disease with spontaneous resolution around puberty [2–6]. 

## Data Availability

Not applicable.

## Abbreviations

VKC: Vernal keratoconjunctivitis
